# Supplementary material for: Low-Temperature Hydrothermal Growth of ZnO Nanowires on AZO Substrates for FACsPb(IBr)3 Perovskite Solar Cells
Source: Nanomaterials (Basel). 2022 Jun 17;12(12):2093. doi: 10.3390/nano12122093 (PMC9229726; doi:10.3390/nano12122093)
Supplement: Supplementary file 1 [file nanomaterials-12-02093-s001.zip › nanomaterials-1758104-supplementary.pdf]

nanomaterials

MDPI

Supplementary information

Low-Temperature Hydrothermal Growth of ZnO Nanowires on AZO Substrates for FACsPb(IBr)3 Perovskite Solar Cells

Karthick Sekar[1,2,3,*], Rana Nakar [1], Johann Bouclé [2,3], Raphaël Doineau [1], Kevin Nadaud [1], Bruno Schmaltz [4], and Guylaine Poulin-Vittrant [1,*]

[1] GREMAN UMR 7347, Université de Tours, CNRS, INSA Centre Val de Loire, 37071 Tours, cedex 2, France; rana.nakar@univ-tours.fr (R.N.); raphael.doineau@univ-tours.fr (R.D.); kevin.nadaud@univ-tours.fr (K.N.)
[2] Univ. Limoges, XLIM, UMR 7252, 87000 Limoges, France.
[3] CNRS, XLIM, UMR 7252, 87000 Limoges, France; johann.boucle@unilim.fr
[4] PCM2E EA 6299, Université de Tours, Parc de Grandmont, 37200 Tours, France; bruno.schmaltz@univ-tours.fr
* Correspondence: karthick.sekar@univ-tours.fr (S.K.); guylaine.poulin-vittrant@univ-tours.fr (G.P.-V.)

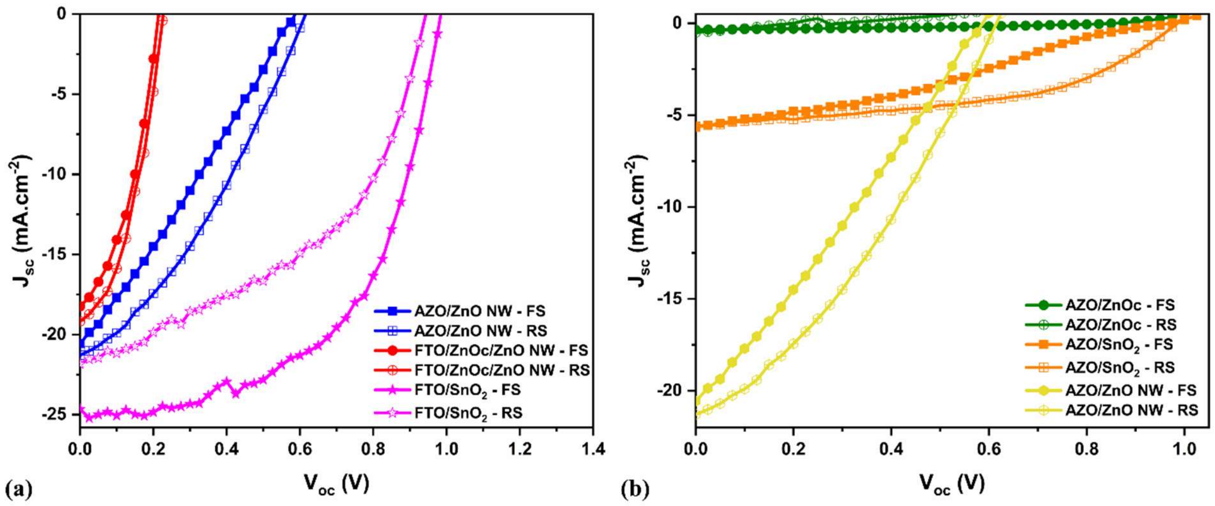

Figure S1. J–V characteristics of perovskite solar cells (best device) fabricated with different photoanodes of ZnO NWs depending on the seed layer. (a) comparison of AZO/ZnO NW, FTO/ZnOc/ZnO NW, and FTO/SnO_2 devices, (b) Comparison of AZO/ZnOc, AZO/SnO_2, and AZO/ZnO NW based devices, respectively.

Table S1. Performances of perovskite solar cells depending on the type of photoanodes.

Device	Scan		Jsc (mA.cm^{-2})	Voc (V)	FF	PCE (%)	R_{sh} (Ω)	R_s (Ω)
AZO/SnO_2/Perovskite/ Spiro-OMeTAD/Au	FS	Best	5.6	0.95	0.31	1.7	1340	780
		Average	5.3	0.95	0.25	1.3	1060	960
	RS	Best	5.6	0.98	0.49	2.7	2210	250
		Average	5.3	0.98	0.47	2.4	2910	410
AZO/ZnOc/Perovskite/ Spiro-OMeTAD/Au	FS	Best	0.3	0.49	0.45	0.1	21940	2840
		Average	0.4	0.84	0.35	0.1	12990	3340
	RS	Best	0.3	0.27	0.43	0.1	13860	1670
		Average	0.5	0.21	0.28	0.1	2440	2370
AZO/ZnO NW/Perovskite/Spiro-OMeTAD/Au	FS	Best	15.6	0.65	0.43	4.3	660	80
		Average	16.4	0.66	0.36	3.8	430	110
	RS	Best	16.1	0.64	0.47	4.9	1400	70
		Average	16.7	0.68	0.43	4.8	1350	80
	FS	Best	16.7	0.20	0.45	1.6	150	20

**FTO/ZnOc/ZnO NW/Perovskite/Spiro-OMeTAD/Au**		Average	14.6	0.19	0.45	1.3	130	30
	RS	Best	17.9	0.21	0.44	1.7	220	20
		Average	17.9	0.20	0.43	1.5	170	20
**FTO/SnO_2/Perovskite/Spiro-OMeTAD/Au**	FS	Best	24.9	0.98	0.56	13.8	19960	40
		Average	24.1	0.97	0.57	13.3	9470	40
	RS	Best	21.9	0.94	0.45	9.3	520	50
		Average	21.6	0.93	0.44	9.1	700	50

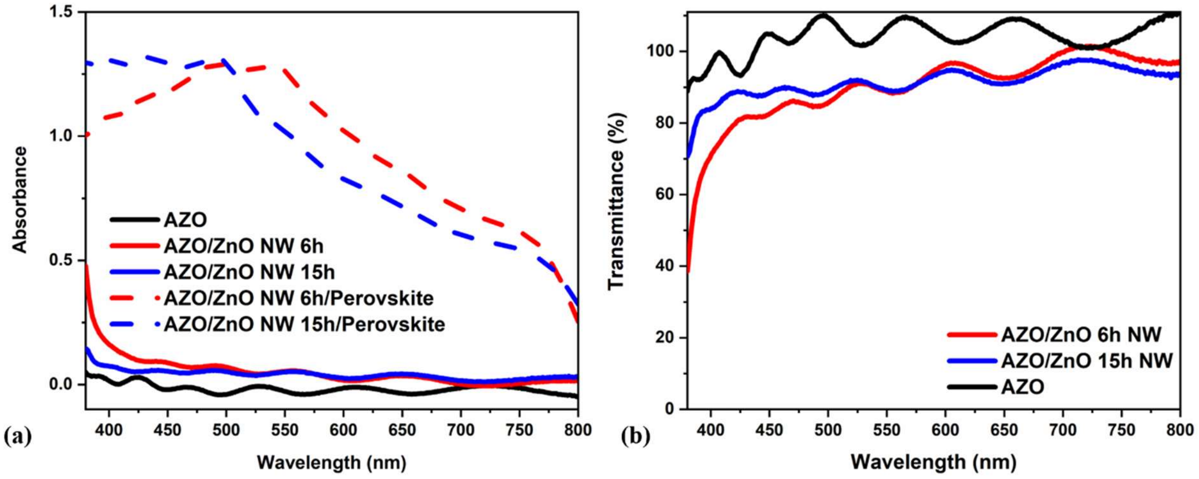

Figure S2. Recorded UV-visible absorbance (a) and transmittance (b) spectral response for ZnO NWs (6h and 15 h) grown on AZO substrate.

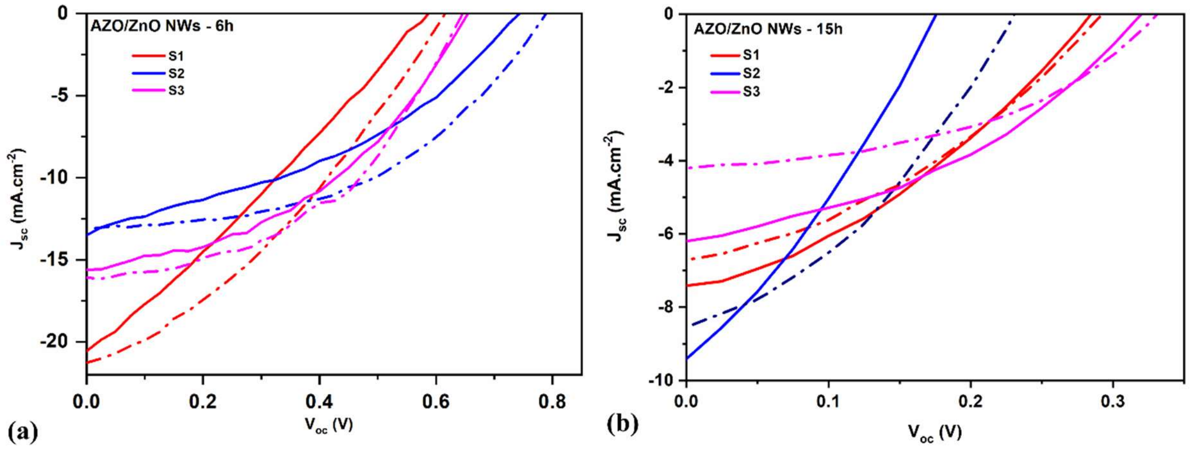

Figure S3. Comparison of several same batch device J-V characteristics of (a) AZO/ZnO NW (6h) and (b) AZO/ZnO NW (15h) solar cells, respectively.

Table S2. Photovoltaic parameters of perovskite solar cells fabricated as a function of ZnO NW array growing time (i.e., 6h and 15h).

Growth time	Device		J_{sc} (mA.cm^{-2})	Voc (V)	FF	PCE (%)	R_{sh} (Ω)	R_s (Ω)
**6h**	1	FS	20.2	0.58	0.28	3.3	200	110
		RS	20.9	0.61	0.34	4.4	460	90
	2	FS	13.4	0.74	0.38	3.7	420	120
		RS	13.1	0.79	0.48	4.9	2180	90
	3	FS	15.6	0.65	0.43	4.3	650	80
		RS	16.1	0.64	0.47	4.9	1400	70
**Average**		FS	16.4	0.66	0.36	3.8	430	110
		RS	16.7	0.68	0.43	4.7	1350	80
**15h**	1	FS	7.0	0.28	0.37	0.7	460	110
		RS	6.5	0.29	0.38	0.7	560	110

	2	FS	8.4	0.17	0.35	0.5	130	70
		RS	7.9	0.23	0.40	0.7	380	70
	3	FS	6.1	0.31	0.40	0.8	560	100
		RS	4.2	0.32	0.46	0.6	1210	130
**Average**		FS	7.2	0.25	0.37	0.7	380	100
		RS	6.2	0.28	0.41	0.7	720	110

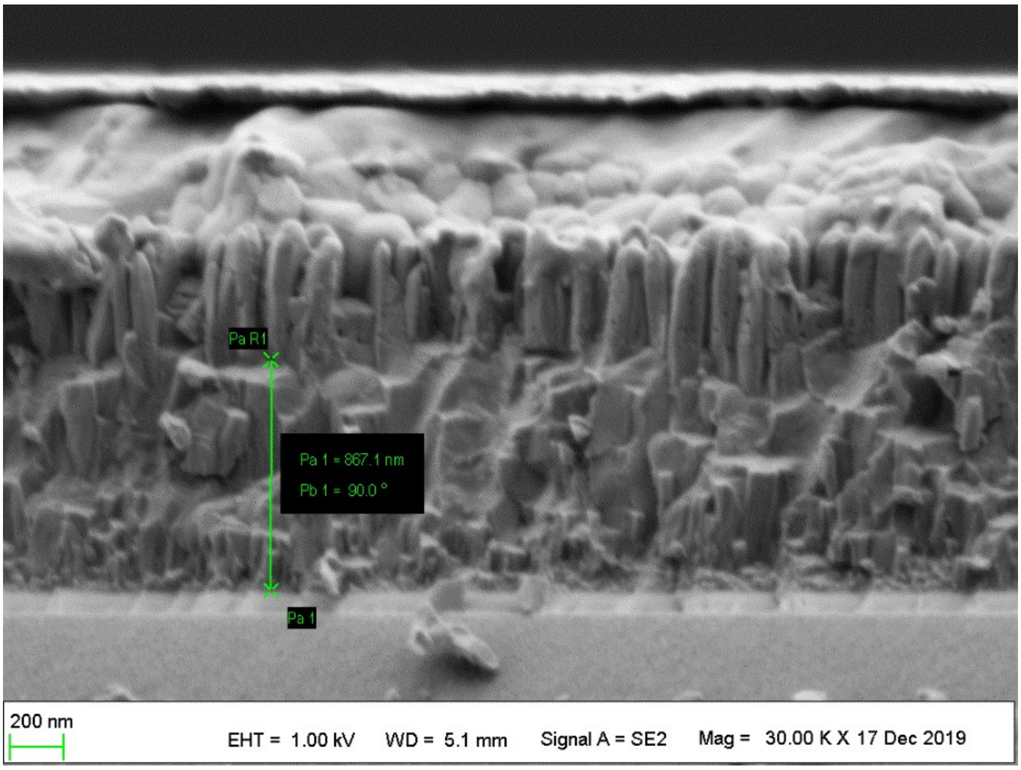

Figure S4. SEM cross-section of a full device based on AZO/ZnO-NW grown for 6h. The image demonstrates a homogeneity of the sandwich structure, with a perovskite solution residing in the ZnO NW layer.

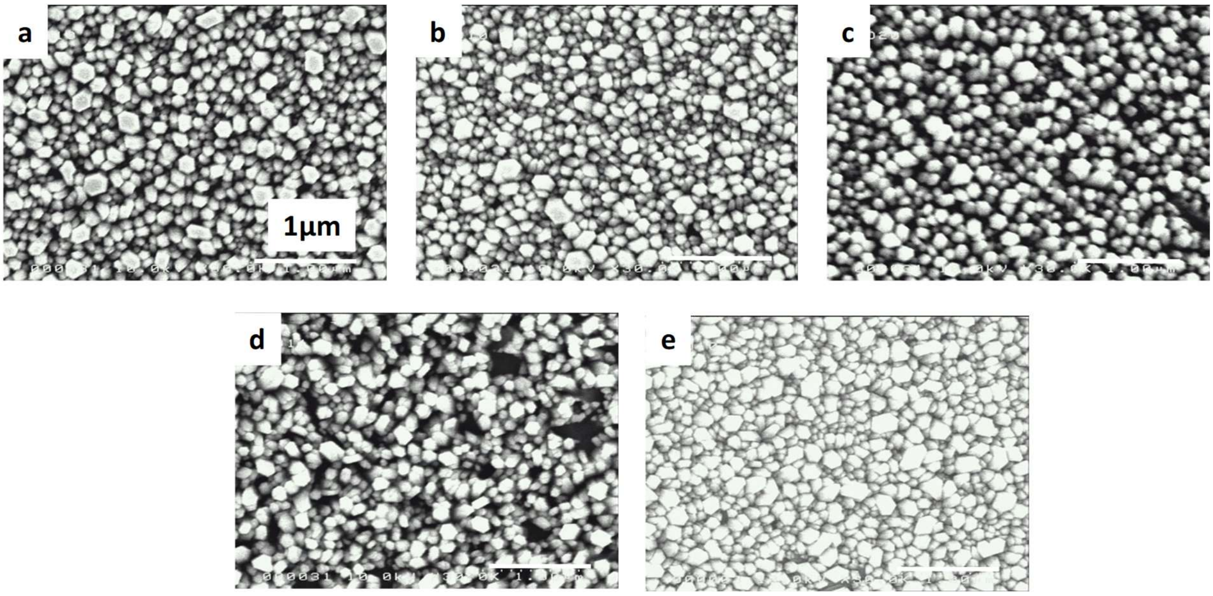

Figure S5. SEM micrographs of ZnO NWs as a function of the [Zn(NO_3)$_2$]/[PEI] ratio, (a) without PEI, (b) 10, (c) 7.15, (d) 4.5, and (e) 3.85, respectively.

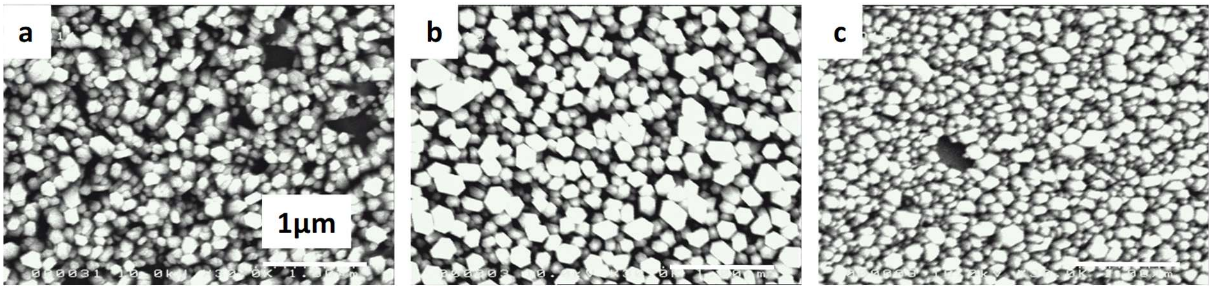

Figure S6. SEM micrographs of ZnO NWs as a function of the [$Zn(NO_3)_2$]/ [HMTA] ratio : (a) 1 ; (b) 1.33 ; (c) 2. The concentration of the $Zn(NO_3)_2$ solution is kept constant at 50mM in all cases, and the growing time is fixed at 6h.

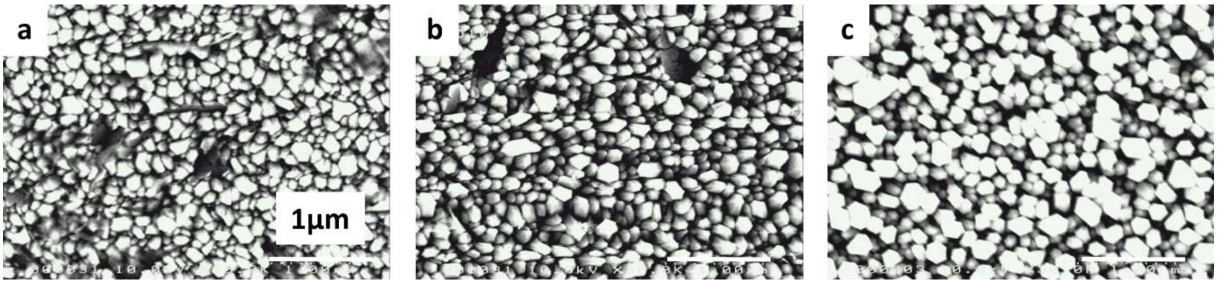

Figure S7. SEM micrographs of ZnO NWs as a function of the [$Zn(NO_3)_2$] (a) 20 mM, (b) 35 mM, and (c) 50 mM, respectively.

Table S3. Summary table of the length, diameter, apparent density, and void fraction of ZnO NWs as a function of different conditions.

	C_1 Zn mM	C_2 HMTA mM	C_1/C_2	C_3 PEI mM	C_1/C_3	L (µm)	d (nm)	D NW/µm^2	Void fraction %
**Zn (50mM), HMTA, PEI (1,1,300). 6h**	50	50	1	5	300	0.4	127	53	~28
**Zn (50mM), HMTA, PEI (1,1,300). 15h**	50	50	1	5	300	1.3	165	29	~58
**Zn (50mM), HMTA, (1,1). 6h**	50	50	1	--	--	0.3	131	59	~36
**Zn (50mM), HMTA, PEI (1,1,10). 6h**	50	50	1	5	10	0.3	135	59	~37
**Zn (50mM), HMTA, PEI (1,1,7.15). 6h**	50	50	1	7	7.15	0.4	147	49	~44
**Zn (50mM), HMTA, PEI (1,1,4.5). 6h**	50	50	1	11	4.5	0.5	~125	58	~45
**Zn (50mM), HMTA, PEI (1,1,3.85). 6h**	50	50	1	13	3.85	0.2	150	50	~28
**Zn (50mM), HMTA, PEI (1,1.33,4.5). 6h**	50	37.5	1.33	11	4.5	0.5	125	58	~36
**Zn (50mM), HMTA, PEI (1,2,4.5). 6h**	50	25	2	11	4.5	0.3	101	76	~37
**Zn (35mM), HMTA, PEI (1,1.33,4.5). 6h**	35	26.6	1.33	7.77	4.5	0.3	117	57	~46
**Zn (20mM), HMTA, PEI (1,1.33,4.5). 6h**	20	15	1.33	4.44	4.5	0.25	109	58	~43

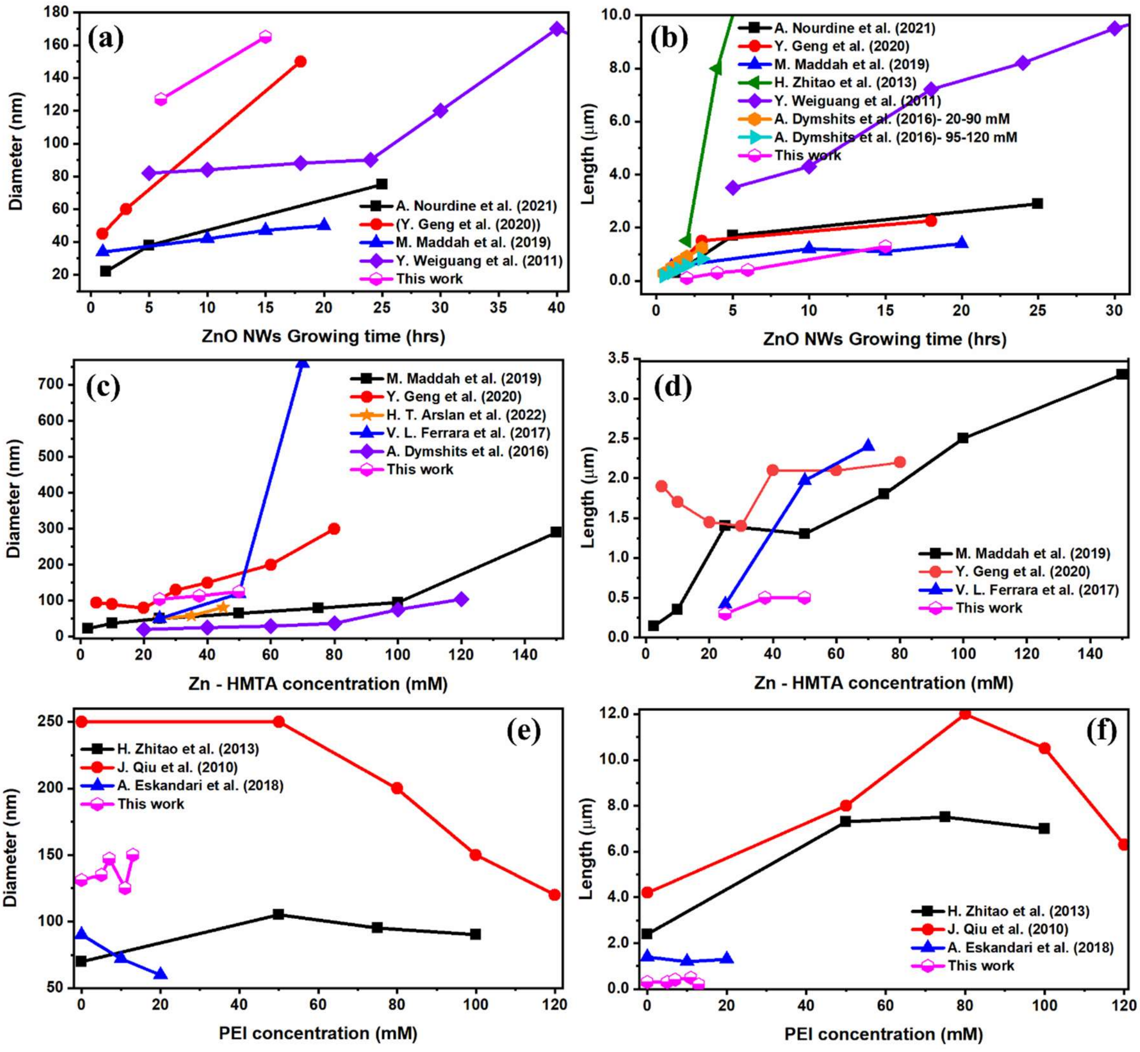

Figure S8. Published reports related to the ZnO NW properties, especially NW length and diameters. Comparison of NW diameters and lengths (a and b) based on growing time, (c and d) based on Zn – HMTA concentration, and (e and f) based on PEI concentration, respectively.

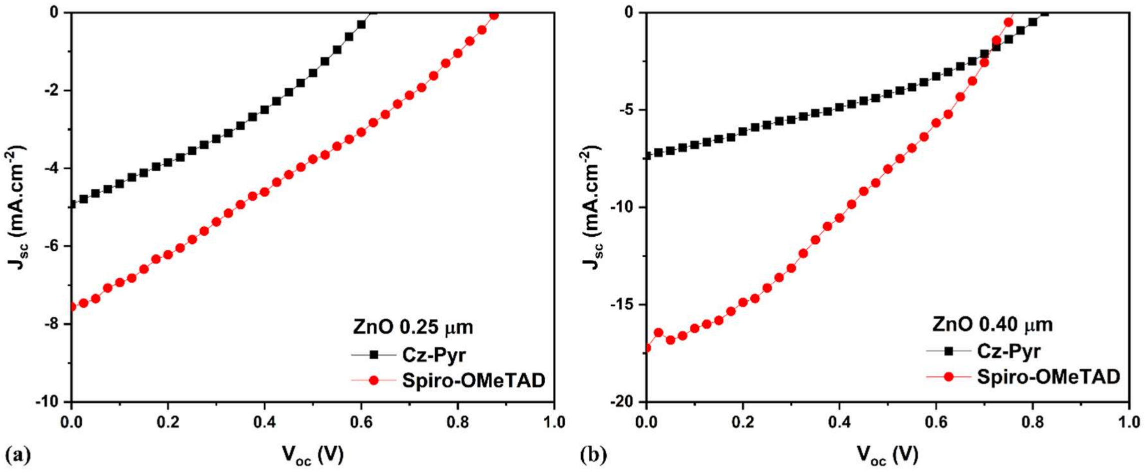

Figure S9. J–V characteristics of perovskite solar cells (best device) fabricated with two different HTLs (Spiro-OMeTAD and Cz-Pyr) using two different NWs (a) ZnO 0.25 µm and (b) ZnO 0.40 µm, respectively.

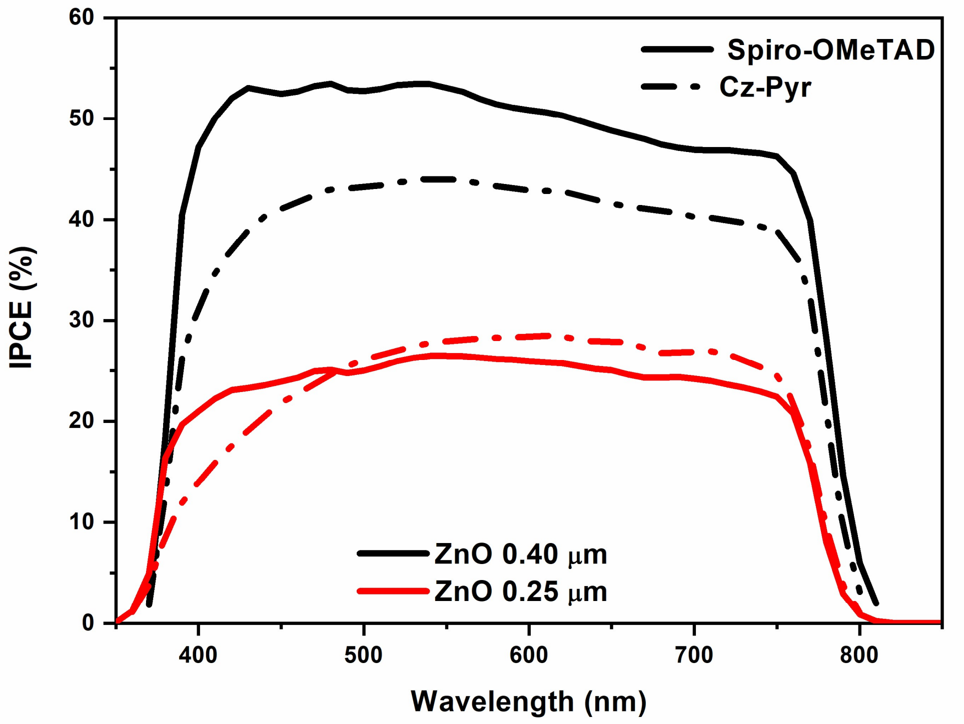

Figure S10. IPCE spectra for the champion solar cells with two different NWs based devices (**ZnO 0.40 μm** and **ZnO 0.25 μm**) using two different HTLs (Spiro-OMeTAD and Cz-Pyr), respectively.

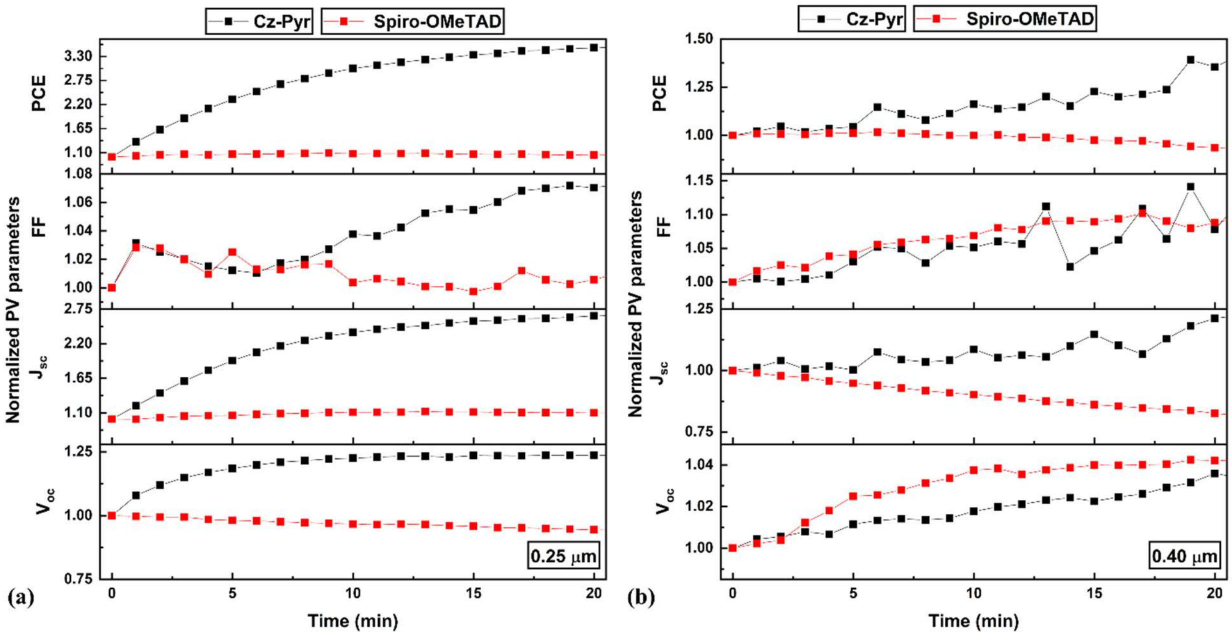

Figure S11. Preliminary aging test of the ZnO NWs (**ZnO 0.25 μm (a) and 0.40 μm (b)**) based devices along with two different HTLs (Spiro-OMeTAD and Cz-Pyr), respectively.
